# Correlation between Deep Venous Thrombosis and Inflammation in Patients after Implantation of Permanent Pacemaker

**Published:** 2020-01

**Authors:** Jianxin MA, Lian CUI, Wenjin HUO, Guanghui WANG, Xin QUAN, Jinping ZHANG

**Affiliations:** 1 Department of Health Care, 305 Hospital of Chinese PLA, Beijing 100017, P.R. China; 2 Department of Echocardiography, Fuwai Hospital, Peking Union Medical College, Beijing 100017, P.R., China; 3 Medical Department, 305 Hospital of Chinese PLA, Beijing 100017, P.R. China

**Keywords:** Cardiac pacemaker implantation, Deep venous thrombosis, Inflammatory factors

## Abstract

**Background:**

The correlation between postoperative deep venous thrombosis (DVT) and inflammation in patients with permanent cardiac pacemaker implantation was analyzed.

**Methods:**

A total of 130 cases undergoing permanent pacemaker implantation in the 305 Hospital of Chinese PLA and Fuwai Hospital from May 2014 to February 2017 were selected. Of the 130 cases, 60 patients complicated with DVT were selected as the observation group, and the remaining 70 cases without complications of DVT were selected as the control group. The relationship and influence of various factors were explored.

**Results:**

The number of patients smoking and the number of subjects with DVT history in the observation group were higher than those in the control group. In the observation group, plasminogen activator inhibitor (PAI)-1: Ag, PAI-1: Ac, thrombin-activated fibrinolysis inhibitor (TAFI): Ag, and TAFI: Ac levels were higher than those in control group (*P*<0.05). The levels of inflammatory factors of the peripheral blood of the observation group were significantly higher than those of the control group (*P*<0.05). In the correlation analysis of serum inflammatory factors and coagulation factors, CRP, IL-6, IL-10 were positively correlated with PAI-1: Ag level. Age, BMI, smoking history, number of implanted electrodes, DVT history, duration of immobilization and inflammatory factor levels had independent predictive value on postoperative complicated DVT.

**Conclusion:**

The serum inflammatory factors are closely associated with postoperative DVT in patients implanted with permanent cardiac pacemaker, and the serum inflammatory factors are a good reference for the evaluation of DVT.

## Introduction

Clinically, the application of permanent pacemaker implantation is increasingly common, especially in patients with arrhythmia (1), but clinicians cannot overcome a series of complications occurring after the permanent pacemaker implantation, such as postoperative infection, implant electrode shedding, deep venous thrombosis (DVT), which affect prognosis and even lead to death (2). Since the permanent pacemaker implantation was put into clinical use, it has been applied from indications of arrhythmia to long QT syndrome, heart failure, vagal dizziness (3).

Regarding complications, infection and DVT are the most serious. For patients with DVT, pulmonary embolism may also occur, increasing the mortality rate (4). In most cases, at the initial stage of DVT, no obvious or only slight clinical manifestations have been observed, which have often not been taken seriously or treated promptly (5). Among fibrinolytic systems, plasminogen activator inhibitor (PAI)-1 and thrombin-activated fibrinolysis inhibitor (TAFI) are the major and most important components (6). The PAI-1 and TAFI content increase leads to reduced fibrinolytic activity, and an important factor in DVT is the reduced fibrinolytic activity (7). Overreaction of the inflammatory system increases the risk of DVT because a damage to the venous wall is a key factor in the development of DVT and thrombosis may aggravate the damage to the vascular wall, especially in the veins, and elevated levels of inflammatory cytokines in peripheral blood are a risk factor for the development of DVT (8,9).

Therefore, analyzing the relationship between inflammation and DVT is helpful to reduce the risk of developing DVT in patients with permanent pacemaker implantation, or to achieve early treatment and improve prognosis.

## Patients and Methods

### General information

A total of 130 cases undergoing permanent pacemaker implantation in 305 Hospital of Chinese PLA (Beijing, China) and Fuwai Hospital (Beijing, China) from May 2014 to February 2017 were selected. Among them, 60 cases with DVT were selected as the observation group, and the other 70 patients without DVT as control group, including 83 males and 47 females, aged 68–78 years, with an average age of 73.73±10.71 years. All patients were selected after permanent pacemaker implantation.

Patients with DVT were diagnosed according to ultrasound and clinical manifestations, by applying pressure on the limbs using a probe and comparing with normal limbs. DVT showed obvious expansion of the lumen, no echo or low echo in the lumen. The lumen cannot be compressed or thrombus can be seen moving in the blood vessels. Exclusion criteria: long-term pre-operative immobilization; systemic infection; the use of estrogen or anticoagulant, thrombolytic drugs; malignant tumor; autoimmune diseases; recent surgery or severe trauma; severe liver and kidney dysfunction; mental illness; clinical data mismatched or refused to sign informed consent. The study was approved by the Ethics Committee of 305 Hospital of Chinese PLA. Informed consents were signed by the patients or guardians.

### Methods

Clinical data from all patients were retrospectively analyzed, including age, sex, BMI, smoking history, the number of electrodes implanted, and history of DVT. Fasting peripheral blood was drawn 10h after fasting for 10 days from all enrolled cases. Biochemical indexes were determined by detecting the upper serum. The data of total cholesterol (TC), triglyceride (TG), LDL-C and HDL-C were measured by an automatic biochemical analyzer provided by Hitachi, Ltd. (Tokyo, Japan). The levels of inflammatory cytokines such as CRP, IL-10 and IL-6 were measured by an immunoturbidimetric assay. PAI-1: Ac, TAFI: Ag, TAFI: Ac levels were measured by enzyme-linked immunosorbent assay (ELISA).

### Statistical methods

SPSS 19.0 statistical software (IBM Corp., Armonk, NY, USA) was used to process the data. The data were expressed as x±s. Pearson correlation analysis was used for the calculation of the r-values between serum inflammatory and clotting factors. Logistic regression analysis was used to examine risk factors for DVT. *P*<0.05 was considered statistically significant.

## Results

### Comparison of general information between observation and control group

There was no significant difference between the observation and control group in terms of sex, but the age, BMI, number of patients smoking, number of electrodes implanted, number of patients with DVT history in the observation group were higher than that of the control group; and the immobilization time of the observation group was also longer than that of the control group. The differences were statistically significant (*P*<0.05) (Table 1).

**Table 1:** Comparison of general information between observation and control group

***General information***	***Observation(n=60)***	***Control(n=70)***	**P-*value***
Age (yr)	77.63±5.38	69.21±7.16	0.043
Sex (f/m)	38/22	45/25	0.793
BMI (kg/m^2^)	30.06±2.82	27.74±2.88	0.039
Smoking history (yes/no)	48/12	30/40	0.028
No. of electrodes implanted	4.52±1.80	1.40±0.39	0.003
DVT history (yes/no)	45/15	23/47	0.001
Postoperative immobilization time (h)	75.17±4.80	48.39±4.65	0.001
DVT, deep venous thrombosis.			

### Comparison of coagulation factor levels

The levels of PAI-1: Ag, PAI-1: Ac, TAFI: Ag, TAFI: Ac in the observation group were higher than those in the control group and the differences were statistically significant (*P*<0.05) (Table 2).

**Table 2:** Comparison of clotting factor levels in observation and control group

***Index***	***Observation (n=60)***	***Control (n=70)***	**P-*value***
PAI-1:Ag (ng/ml)	44.39±4.68	27.67±2.52	0.002
PAI-1:Ac (AU/ml)	0.75±0.29	0.49±0.28	0.017
TAFI:Ag (%)	108.53±22.41	93.73±25.31	0.001
TAFI:Ac (μg/ml)	33.93±7.37	15.51±5.42	0.001

PAI, plasminogen activator inhibitor; TAFI, thrombin-activated fibrinolysis inhibitor

### Comparison of biochemical indicators between the observation and control group

Compared between the two groups, the levels of TC, TG and LDL-C in the observation group were higher than those in the control group, but the differences were not statistically significant (Table 3).

**Table 3:** Biochemical indicators of the observation and control group (μmol/l)

	***Observation***	***Control***	P*-value*
Index	(n=60)	(n=70)	
TC	4.88±0.75	4.21±0.39	0.291
TG	1.87±0.68	1.84±0.59	0.197
LDL-C	2.74±0.61	2.68±0.65	0.509
HDL-C	1.40±0.48	1.42±0.47	0.372

TC, total cholesterol; TG, triglyceride.

### Serum inflammatory factors in observation and control group

The inflammatory factors (CRP, IL-6, IL-10) in peripheral blood serum of patients in the observation group were significantly higher than those in the control group and the differences were statistically significant (*P*<0.05) (Table 4).

**Table 4:** Comparison of serum levels of inflammatory factors in observation and control group

***Index***	***Observation (n=60)***	***Control (n=70)***	**P-*value***
CRP (mg/l)	28.39±2.23	3.32±1.49	0.001
IL-6 (pg/ml)	13.56±5.37	3.02±1.70	0.001
IL-10 (pg/ml)	2.96±1.42	0.91±0.56	0.019

### Correlation analysis of serum inflammatory and clotting factors

**Fig. 1:**
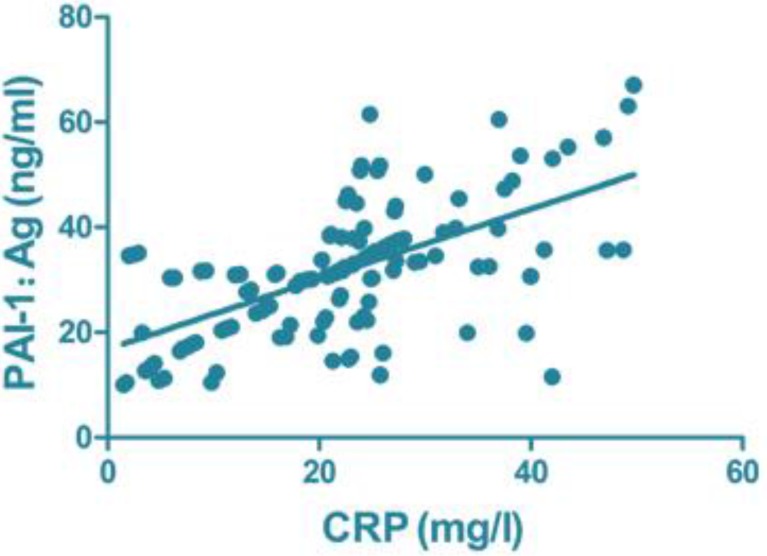
Correlation of CRP with PAI-1: Ag. PAI, plasminogen activator inhibitor

**Fig. 2:**
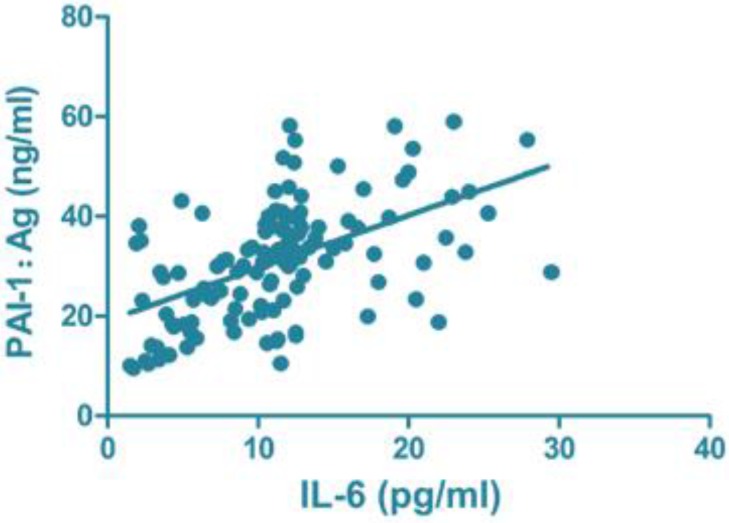
Correlation of IL-6 with PAI-1: Ag. PAI, plasminogen activator inhibitor

**Fig. 3:**
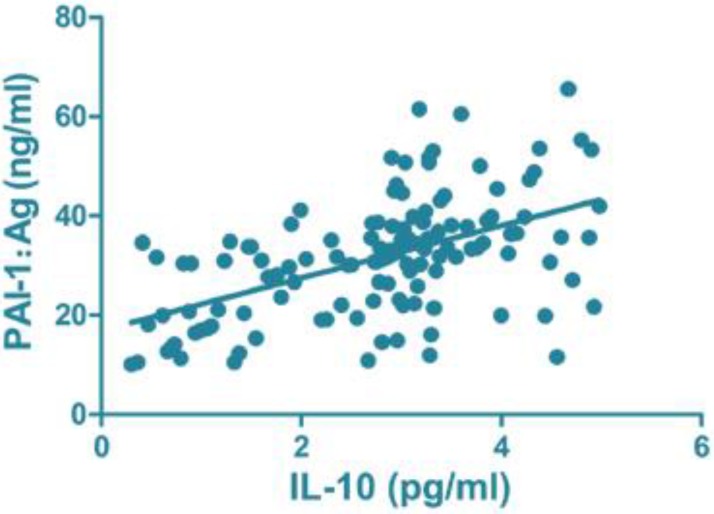
Correlation of IL-10 with PAI-1: Ag. PAI, plasminogen activator inhibitor

CRP (r=0.796, *P*<0.001), IL-6 (r=0.664, *P*<0.001) and IL-10 (r=0.731, *P*<0.001) were all positively correlated with PAI-1: Ag level (Figs. 1–3).

### Logistic regression analysis of risk factors for DVT after permanent pacemaker implantation.

Age, BMI, smoking history, the number of implanted electrodes, the history of DVT, the post- operative immobilization time and the levels of inflammatory factors had independent predictive value on postoperative complicated DVT (Table 5).

**Table 5:** Logistic regression analysis of risk factors for DVT after permanent pacemaker implantation

***Factor***	***P-value***	***OR-value***	***95% CI***
Age	0.018	1.095	0.968–1.164
Sex	0.537	0.992	0.105–6.672
BMI	0.004	5.435	1.923–18.151
Smoking history	0.027	6.538	1.919–15.146
No. of implanted electrodes	0.017	1.432	0.577–3.574
DVT history	0.031	7.084	1.936–8.679
Postoperative immobilization time	0.037	1.793	0.962–7.143
TC	0.521	1.459	0.467–4.494
TG	0.057	1.449	0.763–7.147
LDL-C	0.052	2.742	0.964–7.138
HDL-C	0.952	0.079	0.451–18.714
CRP	0.001	1.003	0.979–1.045
IL-6	0.023	1.058	0.969–1.218
IL-10	0.019	1.035	0.985–1.036

DVT, deep venous thrombosis; TC, total cholesterol; TG, triglyceride.

## Discussion

Clinically, permanent pacemakers are widely used in patients with cardiac arrhythmias by generating and releasing a relative amount of magnetic impulses that interconnect with the patient's cardiac tissue cells (10). After receiving the pulse wave, the myocardial cells can affect the surrounding myocardial tissue cells and reconduct the pulse wave so that all heart cells can receive a certain degree of stimulation, resulting in normal heart diastolic and systolic exercise, which is the main mechanism of treating patients by permanent pacemaker (11).

Although pacemakers are unable to replace cardiomyocytes for normal systolic and diastolic activity, they can mainly affect cardiomyocytes through stimulations for therapeutic purposes (12). In patients with arrhythmia, permanent pacemaker implantation is prone to lead to some common complications, such as infection, DVT; these complications are critical and will even lead to death (13). After operation, the release of coagulation factors is disturbed and further formation of thrombus is promoted because the implanted electrodes damage the blood vessel wall tissue cells to varying degrees (14). Furthermore, the contact between the electrode and the original lesion in the thrombus also increases the probability of DVT (15). After a permanent pacemaker implantation, DVT tends to occur because the patient has an excessive BMI and prolonged postoperative immobilization due to the patient's blood flow slowing down when the patient is immobilized for an extended period of time, DVT is likely to occur (16). The age and BMI of the observation group was greater than that in the control group, the number of electrodes implanted and the number of patients with DVT history were more than those in the control group, and postoperative immobilization time was also longer than that in the control group (*P*<0.05), which was consistent with the above conclusion.

In the development and progression of DVT, activators such as plasminogens are one of the most dangerous factors and are associated with an overreaction with the inflammatory system (17). In the blood system, the most important reason for stimulating fibrinolytic time is thrombin, and PAI-1, TAFI-1 are closely related with fibrinolysis time and they are major markers of fibrinolysis (18). PAI-1, TAFI-1, which are mainly expressed as PAI-1: Ac, TAFI-1: Ac after activation, they are the main force of fiber dissolution (19). The PAI-1: Ag, PAI-1: Ac, TAFI: Ag, TAFI: Ac levels in patients with DVT after permanent pacemaker implantation were significantly higher than those patients without DVT, and the difference was statistically significant (*P*<0.05).

The disorder of the inflammatory system can lead to or aggravate the formation of DVT, and DVT can further promote the inflammatory reaction of the blood vessel wall, resulting in a vicious circle (8). The activation and continual regulation of tissue factor affect the inflammatory system, and the increased level of inflammatory factors in peripheral blood is a high-risk factor of DVT (9). Large amounts of data have shown that CRP, IL-6 and IL-10 levels and other inflammatory cytokine levels in the peripheral blood serum of patients with DVT are increased up to nearly eight times, and it has been suggested that DVT itself is accompanied by overreaction of the inflammatory system (20). The present study found that the levels of peripheral inflammatory factors in the observation group were higher than those in the control group, and the correlation analysis between serum inflammatory factors and blood coagulation factors showed that the levels of CRP, IL-6, IL-10 and PAI-1:Ag level were positively correlated, indicating that the higher the level of inflammatory cytokines in serum, the greater the risk of DVT, which is consistent with most of the findings.

## Conclusion

To explore the relationship between DVT and inflammation in patients after permanent pacemaker implantation can reduce the incidence of DVT, or provide early intervention in the early stage of disease to improve the prognosis and reduce mortality.

## Ethical considerations

Ethical issues (Including plagiarism, informed consent, misconduct, data fabrication and/or falsification, double publication and/or submission, redundancy, etc.) have been completely observed by the authors.
